# Searching for transcription factor binding sites in vector spaces

**DOI:** 10.1186/1471-2105-13-215

**Published:** 2012-08-27

**Authors:** Chih Lee, Chun-Hsi Huang

**Affiliations:** 1 Department of Computer Science and Engineering, University of Connecticut, Fairfield Road, Storrs, CT 06269, USA

## Abstract

**Background:**

Computational approaches to transcription factor binding site identification have been actively researched in the past decade. Learning from known binding sites, new binding sites of a transcription factor in unannotated sequences can be identified. A number of search methods have been introduced over the years. However, one can rarely find one single method that performs the best on all the transcription factors. Instead, to identify the best method for a particular transcription factor, one usually has to compare a handful of methods. Hence, it is highly desirable for a method to perform automatic optimization for individual transcription factors.

**Results:**

We proposed to search for transcription factor binding sites in vector spaces. This framework allows us to identify the best method for each individual transcription factor. We further introduced two novel methods, the negative-to-positive vector (NPV) and optimal discriminating vector (ODV) methods, to construct query vectors to search for binding sites in vector spaces. Extensive cross-validation experiments showed that the proposed methods significantly outperformed the ungapped likelihood under positional background method, a state-of-the-art method, and the widely-used position-specific scoring matrix method. We further demonstrated that motif subtypes of a TF can be readily identified in this framework and two variants called the *k* NPV and *k* ODV methods benefited significantly from motif subtype identification. Finally, independent validation on ChIP-seq data showed that the ODV and NPV methods significantly outperformed the other compared methods.

**Conclusions:**

We conclude that the proposed framework is highly flexible. It enables the two novel methods to automatically identify a TF-specific subspace to search for binding sites. Implementations are available as source code at: http://biogrid.engr.uconn.edu/tfbs_search/.

## Background

Transcription of genes followed by translation of their transcripts into proteins determines the type and functions of a cell. Expression of certain genes even initiates or suppresses differentiation of stem cells. It is therefore crucial to understand the mechanisms of transcriptional regulation. Among them, transcription factor (TF) binding is the one that has been given considerable attention by computational biologists for the past decade and is still being actively researched. A TF is a protein or protein complex that regulates transcription of one or more genes by binding to the double-stranded DNA. A first step in computational identification of target genes regulated by a TF is to pinpoint its binding sites in the genome. Once the binding sites are found, the putative target genes can be searched and located in flanking regions of the binding sites.

In general, there are two approaches to computational transcription factor binding site (TFBS) identification, motif discovery and TFBS search. The former assumes that a set of sequences is given and each of the sequences may or may not contain TFBSs. An algorithm then predicts the locations and lengths of TFBSs. The term motif refers to the pattern that are shared by the discovered TFBSs. These algorithms rely on no prior knowledge of the motif and hence are known as *de novo* motif discovery algorithms. The latter assumes that, in addition to a set of sequences, the locations and lengths of TFBSs are known. An algorithm then learns from these examples and predicts TFBSs in new sequences. Such algorithms are also called supervised learning algorithms since they are guided by the given sequences with known TFBSs. Plenty of efforts have been devoted to the *de novo* motif discovery problem [1-11]. Comprehensive evaluation and comparison of the developed tools have been performed [12,13]. In this study, we focus on the problem of TFBS search. We refer readers interested in the motif discovery problem to the evaluation and review articles [12-14] and references therein.

A typical TFBS search method searches for the binding sites of a particular transcription factor in the following manner. It scans a target DNA sequence and compare each length *l* sub-sequence (*l* -mer) to the binding site profile of the TF, where *l* is the length of a binding site. Each of the *l* -mer is scored when comparing to the profile. A cut-off score is then set by the method to select candidate TF binding sites. The position-specific scoring matrix (PSSM) [15] is a widely used profile representation, where the binding sites of a TF are encoded as a 4 ×* l* matrix. Column *i* of the matrix stores the scores of matching the *i*^th^ letter in an *l* -mer to nucleotides A, C, G and T, respectively. Depending on the method of choice, the score of A at position *i* can be the count of A at position *i* in the known TFBSs, the log-transformed probability of observing A at position *i* , or any other reasonable number. Once computed, the scoring matrix of a TF can be stored in a database. These matrices are used by tools [16-21] to scan sequences for TFBSs.

One assumption the PSSM representation makes is that positions in a binding site are independent, which is often not the case. Osada *et al.*[22] exploited dependence between positions by considering nucleotide pairs in scoring methods. It was shown that incorporating nucleotide pairs significantly improved the performance of a method, meaning that most transcription factors studied demonstrated correlation between positions in a binding site. This result was reinforced in a recent study [23], in which the authors showed correlations between two nucleotides within a binding site by plotting the mutual information matrix. A novel scoring method called the ungapped likelihood under positional background (ULPB) method was proposed in this study. The ULPB method models a TFBS by two first-order Markov chains and scores a candidate binding site by likelihood ratio produced by the two Markov chains. leave-one-out cross-validation results on 22 TFs with 20 or more binding sites showed that ULPB is superior to the methods compared in their work.

In this work, we approach the TFBS search problem from a different perspective. We propose to search for binding sites in vector spaces. Specifically, *l* -mers are placed in the Euclidean space such that each *l* -mer corresponds to a vector in the space. With known binding sites of a TF, we construct a profile vector for the TF. This profile vector can then be used as a query vector to search for the unknown binding sites in the space given a similarity measure between two vectors. The vector space model has long been used in information retrieval (IR) [24,25]. Under this model, each document in a collection is embedded in a *t* -dimensional space. That is, each document is represented by a *t* -element vector, where *t* is the number of distinct terms present in the document collection or corpus. To search for documents on a particular topic, a query composed of terms relevant to the topic is constructed. The query can be similarly embedded in the *t* -dimensional space. Similarity between the query and a document can then be measured by measuring the similarity between the two corresponding vectors. In the TFBS search problem, the entire genome or the collection of promoter region sequences corresponds to the corpus, whereas an *l* -mer is analogous to a document in IR. On the other hand, a TF is analogous to a topic, while a TF representation is the analog of a query for the topic.

In this framework, we propose two novel approaches to constructing a query vector for a TF of interests. We compare the proposed methods to a state-of-the-art method, the ULPB method, as well as the widely-used PSSM method. Performance of a method is assessed by cross-validation experiments on two data sets collected from RegulonDB [26] and JASPAR [27], respectively. Independent validation on human ChIP-seq data gives further insights into the proposed methods. Finally, we discuss the advantages of searching for TF binding sites in the proposed framework.

The paper is organized as follows. In Methods, we present the novel negative-to-positive vector and optimal discriminating vector methods, in addition to introducing the existing methods compared in this work. Cross-validation results on prokaryotic and eukaryotic transcription factors are presented and discussed in Results and Discussion. Finally, we give the concluding remarks in Conclusions.

## Methods

### Data sets

To understand the compared methods in this work, we experimented on prokaryotic as well as eukaryotic transcription factors. The known prokaryotic TF binding sites were collected from from RegulonDB [26] release 6.8. Considered in [23], this data source contains binding sites of TFs in the *E. coli* K-12 genome. We considered a data set of 26 TFs with 17 or more known binding sites. The filtering criterion ensures that, for each TF, we have enough examples to learn from. Similar filtering criteria were used in [23]. This data set is summarized in Table 1.

**Table 1:** Statistics of the E. coli TFs in RegulonDB

**Name**	**Length**	**# TFBSs**	**Name**	**Length**	**# TFBSs**
MetJ	8	29	Lrp	12	62
SoxS	18	19	H-NS	15	37
FlhDC	16	20	AraC	18	20
Fis	15	206	ArcA	15	93
IHF	13	101	OmpR	20	22
PhoB	20	17	GlpR	20	23
OxyR	17	41	CpxR	15	37
NarL	7	90	CRP	22	249
TyrR	18	19	NarP	7	20
Fur	19	81	LexA	20	40
NtrC	17	17	FNR	14	87
MalT	10	20	PhoP	17	21
ArgR	18	32	NsrR	11	37

The known eukaryotic TF binding sites were collected from JASPAR CORE database (the 4^th^ release) [27]. TFs of Homo sapiens and Mus musculus were filtered by two criteria. A TF was kept only if it has at least 20 known binding sites and the length of its binding sites is at least 6 nucleotides. The length criterion, arbitrarily chosen, ensures a TF under consideration is specific enough. This data set is summarized in Table 2.

**Table 2:** Statistics of TFs in the JASPAR database

**Mus musculus**			
**ID**	**Name**	**Length**	**# TFBSs**
MA0039.2	Klf4	10	4336
MA0047.2	Foxa2	12	809
MA0062.2	GABPA	11	87
MA0065.2	PPARG::RXRA	15	839
MA0104.2	Mycn	26	85
MA0141.1	Esrrb	12	3613
MA0142.1	Pou5f1	15	1332
MA0143.1	Sox2	15	666
MA0144.1	Stat3	19	830
MA0145.1	Tcfcp2l1	14	3931
MA0146.1	Zfx	20	477
MA0147.1	Myc	10	682
MA0154.1	EBF1	10	21
**Homo sapiens**			
**ID**	**Name**	**Length**	**# TFBSs**
MA0037	GATA3	6	20
MA0052	MEF2A	10	31
MA0077	SOX9	9	45
MA0080.2	SPI1	7	35
MA0083	SRF	12	26
MA0112.2	ESR1	20	472
MA0115	NR1H2::RXRA	17	22
MA0137.2	STAT1	15	2082
MA0138	REST	19	22
MA0138.2	REST	11	871
MA0139.1	CTCF	11	944
MA0148.1	FOXA1	11	896
MA0149.1	EWSR1-FLI1	17	101
MA0159.1	RXR::RAR_DR5	17	23
MA0258.1	ESR2	18	356

### Notation

For clarity, we list and define functions and variables used throughout this paper. Please see Additional file 1 for more details.

• *f*_*i*_(*u*) denotes the probability of observing letter *u* at position *i* of a TFBS, where *u* ∈{A, C, G, T}.

• *f*_*i*,*j*_(*u*,*v*) denotes the probability of observing letters *u* and *v* at positions *i* and *j* , respectively, where *i* <*j* and *u*,*v* ∈{A, C, G, T}.

• *f*_*i*_(*v*|*u*) denotes the position-specific conditional probability of observing *v* at position *i* + 1 given *u* has been seen at position *i* , where *u* ,*v* ∈{A, C, G, T}.

• *f* (*v*|*u*) denotes the background conditional probability of observing *v* given *u* has been observed at the previous position, where *u* ,*v* ∈{A, C, G, T}.

• $I_{u}(\cdot )$ is the indicator function given by

(1) $$I_{u}(v)=\left\{\begin{array}{cc} 1 & \;\;\text{if}v=u,\\ 0 & \;\text{otherwise,} \end{array}\right)$$

where *u* ,*v* ∈{A, C, G, T}.

• $I_{u_{1}u_{2}}(\cdot )$ is similarly defined as follows:

(2) $$I_{u_{1}u_{2}}(v_{1}v_{2})=\left\{\begin{array}{cc} 1 & \;\;\text{if}\;v_{1}=u_{1}\;\text{and}\;v_{2}=u_{2},\\ 0 & \;\;\text{otherwise,} \end{array}\right)$$

where *u*_1_,*u*_2_,*v*_1_,*v*_2_∈{A, C, G, T}.

• *IC*_*i *_denotes the information content at position *i* of a binding site. Information content is closely related to entropy, a measure of uncertainty in information theory. The entropy at position *i* is given by $E_{i}=-\underset{u\in \{\text{A, C, G, T}\}}{\sum }f_{i}(u){\text{log}}_{2}\left[f_{i}(u)\right]$. When $f_{i}(u)=\frac{1}{4}$ for all *u* ∈{A, C, G, T}, *E*_*i *_attains the maximal entropy of 2 and we are most uncertain about the letter at position *i* . *IC*_*i*_ is simply defined as

(3) $$IC_{i}=2-E_{i}=2+\underset{u\in \{\text{A, C, G, T}\}}{\sum }f_{i}(u){\text{log}}_{2}\left[f_{i}(u)\right].$$

• *IC*_*i*,*j*_ denotes the information content of the position pair (*i* ,*j* ) of a binding site. Similarly,

(4) $$IC_{i,j}=4+\underset{u,v\in \{\text{A, C, G, T}\}}{\sum }f_{i,j}(u,v){\text{log}}_{2}\left[f_{i,j}(u,v)\right],$$

where the maximal entropy of 4 is attained when $f_{i,j}(u,v)=\frac{1}{16}$ for all *u* ,*v* ∈{A, C, G, T}.

### Embedding short sequences in vector spaces

**Figure 1:**
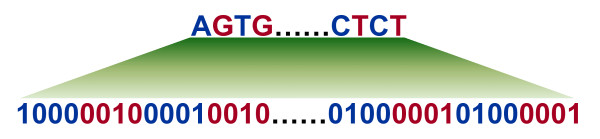
**Illustration of embedding a short sequence in vector space.** Each nucleotide in the sequence is converted to 4 indicator variables.

We describe how a short sequence of *l* nucleotides or an *l* -mer is placed in a vector space. Let *s* be an *l* -mer and *s*_*i*_ denote its *i*^th^ nucleotide. Each nucleotide in *s* is converted to 4 variables, that is, *s*_*i *_is converted to $w_{i}I_{\text{A}}(s_{i}),w_{i}I_{\text{C}}(s_{i}),w_{i}I_{\text{G}}(s_{i})\;\text{and}\;w_{i}I_{\text{T}}(s_{i})$ for *i* = 1,2,…,*l* . Hence, *s* is converted to *4l* variables, placing *s* in $R^{4l}$. Figure 1 illustrates the conversion of each nucleotide in an *l* -mer to 4 variables when *w*_*i *_= 1 for *i* = 1,2,…,*l* .

We further consider nucleotide pair (*s*_*i*_,*s*_*j*_), where *i* <* j* . Only pairs in close proximity are considered in this study. We consider (*s*_*i*_,*s*_*j*_) only if *j* −*i* = 1 or 2, i.e., a pair of nucleotides is considered only if they are adjacent or separated by one nucleotide. Nucleotide pair (*s*_*i*_,*s*_*j*_) is similarly converted to 16 variables, $w_{i,j}I_{\mathrm{AA}}(s_{i}s_{j}),w_{i,j}I_{\mathrm{AC}}(s_{i}s_{j}),\ldots ,w_{i,j}I_{\mathrm{TT}}(s_{i}s_{j})$, as there are 16 possible nucleotide pairs, {AA, AC,…,TT}. We use 32*l* −48 additional variables to encode the pairs since there are *l* −1 adjacent pairs and *l* −2 pairs separated by one nucleotide. Consequently, considering individual nucleotides and nucleotide pairs, each *l* -mer is converted to a (36*l* −48)-element vector.

In this study, we consider two choices of *w*_*i*_’s and *w*_*i*,*j*_’s. For the first choice, all the nucleotides and nucleotide pairs are given the same weight, i.e., *w*_*i *_= 1 and *w*_*i*,*j *_= 1 for all *i* and *j* . The second one assigns weight to the *i*^th^ nucleotide according to the information content at position *i* . Similarly, it assigns weight to pair *i* ,*j*) according to the information content at this pair of positions. Specifically, $w_{i}=\sqrt{IC_{i}}$ and $w_{i,j}=\sqrt{IC_{i,j}}$ for all *i* and *j* .

### Searching for TFBSs in vector spaces

Given a query vector ***t*** in space, we score an *l* -mer *s* as follows:

(5) $$\mathrm{Score}(s)=s^{T}t,$$

where ***s ***denote the corresponding vector of *s* . In other words, the score of *s* is obtained by taking the dot-product between ***s*** and ***t***. It can be seen that Score(*s* ) measures the similarity between ***s ***and ***t***. Assuming that ***t*** corresponds to an *l* -mer *t* , Score(*s* ) counts the number of nucleotides and nucleotide pairs shared between *s* and *t* when *w*_*i *_= 1 and *w*_*i*,*j *_= 1 for all *i* and *j* . However, we note that ***t ***can be any vector in the space and does not necessarily correspond to an *l* -mer.

As described above, an *l* -mer is converted to a (36*l* −48)-element vector. Hence, we use ***t*** to search for binding sites in $R^{\left(36l-48\right)}$. Our approach offers great flexibility in that it easily allows searching for binding sites in a lower dimensional subspace. By setting all but the first *4l* elements in ***t*** to zero, we are essentially searching for binding sites in $R^{4l}$. In this work, we exploit this advantage and simultaneously search for transcription factor binding sites in three subspaces. Two of them are $R^{4l}$ and $R^{\left(36l-48\right)}$. The third one is $R^{\left(16l-12\right)}$. This subspace is obtained from considering only the first nucleotide and the *l* −1 adjacent nucleotide pairs as in a first order Markov chain.

### The NPV method

We first introduce a simple approach to constructing a query vector. Let *P* be the set of *n*_+_ binding sites and *N* be the set of *n*_−_non-binding sites of a particular transcription factor. We embed all the *l* -mers in *P* and *N* in $R^{\left(36l-48\right)}$. We then find the mean binding site vector

$$\mu_{+}=\frac{1}{n_{+}}\underset{s\in P}{\sum }s$$

as well as the mean non-binding site vector

$$\mu_{-}=\frac{1}{n_{-}}\underset{s\in N}{\sum }s.$$

**Figure 2:**
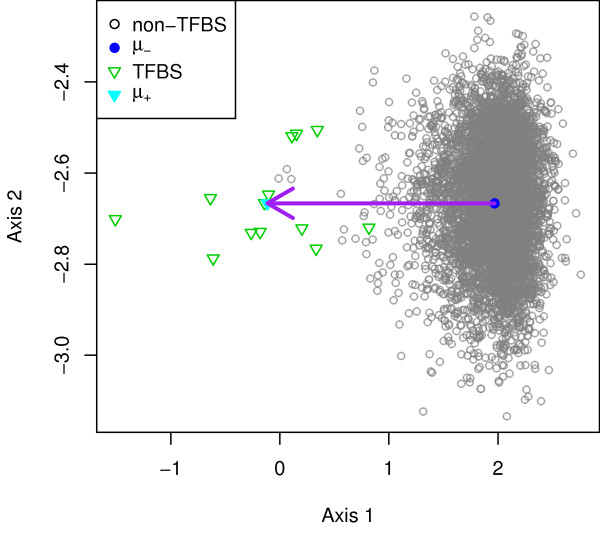
**Illustration of the NPV method.** The solid arrow represents the negative-to-positive vector ***μ***_+_−***μ***_−_, pointing from ***μ***_−_to ***μ***_+_. The hallow triangles denote the known binding sites, whereas the circles represent the known non-binding sites. The center of the binding site vectors is marked by the solid triangle, while the center of the non-binding site vectors is marked by the solid circle.

The query vector ***t*** is found by subtracting ***μ***_−_from ***μ***_+_, that is, ***t ***=*** μ***_+_−***μ***_−_. The query vector ***t ***can be seen as the vector pointing from the center of the non-binding site vectors to the center of the binding site vectors. Hence, we call it the negative-to-positive vector (NPV) method. Figure 2 illustrates the idea.

The score of an *l* -mer *s* given by the NPV method is therefore

(6) $$\mathrm{Score}(s)=s^{T}(\mu_{+}-\mu_{-})=s^{T}\mu_{+}-s^{T}\mu_{-}.$$

We can see that it computes the similarity between *s* and the mean binding site vector as well as the similarity between *s* and the mean non-binding site vector. It then scores *s* by the difference of the two similarity scores. The more similar *s* is to the mean binding site vector, the higher the score. The less similar *s* is to the mean non-binding site vector, the higher the score.

**Figure 3:**
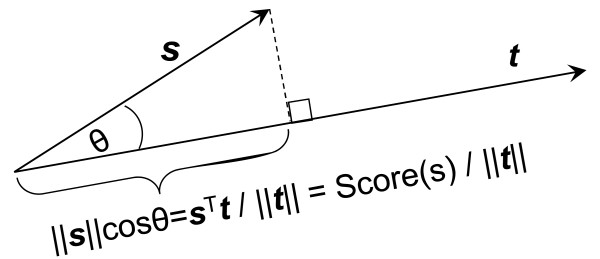
**The orthogonal projection of ***s ***onto ***t***.** It can be seen that the projection of ***s ***onto ***t ***is equal to Score(s)/||t||∝Score(s).

From the perspective of geometry, we note that Score(*s* ) in (5) is proportional to Score(*s* )/||***t***|| , where ||***t***|| is the length of the query vector ***t***. Moreover, by virtue of the equality

$$s^{T}t=||s||\;\;||t||\cos\theta ,$$

we know Score(*s* )/||***t***|| equals the orthogonal projection of ***s ***onto ***t***, where *θ* is the angle formed by vectors ***s*** and ***t*** (see Figure 3 for an illustration). The computation of Score(*s*) is therefore equivalent to computation of the orthogonal projection of ***s*** onto ***t***. Similarly, the computation of Score(*s*) in (6) is equivalent to computation of the orthogonal projection of ***s ***onto ***μ***_+_−***μ***_−_. In Figure 2, we observe that vector ***μ***_+_−***μ***_−_is pointing to the left and, projected onto this vector, most of the binding sites are on the left of the non-binding sites. This implies that most of the binding sites have a higher score than the non-binding sites.

### The ODV method

We have described the NPV method, which offers a heuristic way of constructing a query vector. We now introduce a way of finding an optimal query vector $\beta \in R^{\left(36l-48\right)}$. Suppose that |*P* |=*n*_+_ and |*N* |=*n*_−_, that is, there are *n*_+_ binding sites and *n*_−_ non-binding sites for a particular TF. Let *P* ={*s*_(1)_,*s*_(2)_,…,*s*_(*n*_+_)_} and $N=\{s_{\left(n_{+}+1\right)},s_{\left(n_{+}+2\right)},\ldots ,s_{\left(n\right)}\}$ , where *s*_(*i*)_ denotes the *i*^th^*l* -mer in the union of the two sets and *n* =*n*_+_ + *n*_−_. We find the optimal ***β***by solving the following minimization problem:

(7) $$\begin{array}{ll} \underset{\;\;\;\beta ,b,\xi }{\;\;\;\text{min}} & \;\;\frac{1}{2}||\beta |{|}^{2}+\frac{C}{n_{+}}\overset{n_{+}}{\underset{i=1}{\sum }}\xi_{i}+\frac{C}{n_{-}}\overset{n}{\underset{i=n_{+}+1}{\sum }}\xi_{i}\; \end{array}$$

(8) $$\begin{array}{ll} \text{subject to} & \frac{\mathrm{Score}(s_{\left(i\right)})}{\left||\beta |\right|}\geq \frac{b+1-\xi_{i}}{\left||\beta |\right|}\text{for}s_{\left(i\right)}\in P,\; \end{array}$$

(9) $$\begin{array}{ll} & \;\;\;\;\;\;\frac{\mathrm{Score}(s_{\left(i\right)})}{\left||\beta |\right|}\leq \frac{b-1+\xi_{i}}{\left||\beta |\right|}\text{for}s_{\left(i\right)}\in N,\; \end{array}$$

(10) $$\begin{array}{ll} & \;\;\;\;\;\;\xi_{i}\geq 0\;\;\forall \mathrm{i.}\; \end{array}$$

The constraint in (8) ensures that the projection of a TFBS *s*_(*i*)_ onto the vector ***β***, $\frac{\mathrm{Score}(s_{\left(i\right)})}{\left||\beta |\right|}$, exceeds the threshold $\frac{b+1}{\left||\beta |\right|}$. On the other hand, the constraint in (9) ensures that the projection of a non-TFBS *s*_(*i*)_ onto ***β*** stays below the threshold $\frac{b-1}{\left||\beta |\right|}$. Flexibility is given to the thresholds by introducing *ξ*_*i*_’s with cost captured by the last two terms in (7). Finally, to clearly distinguish TFBSs from non-TFBSs, the squared difference between the two thresholds ( $\frac{b+1}{\left||\beta |\right|}$ and $\frac{b-1}{\left||\beta |\right|}$) is made as large as possible. This amounts to maximizing ${\left(\frac{2}{\left||\beta |\right|}\right)}^{2}$ or, equivalently, minimizing $\frac{1}{2}||\beta |{|}^{2}$, which is the first term in (7). We call this approach the optimal discriminating vector (ODV) method.

The optimization problem in (7) is known as a quadratic programming problem with linear inequality constraints specified in (8), (9) and (10). There are *p* + *n* + 1 variables and *2n* constraints, where *p* =36*l* −48 is the dimension of ***β***. We can see that (8) and (9) specify *n* constraints whereas (10) imposes *n* constraints on the variables. Quadratic programming [28] is well-studied and hence general solvers are available, e.g., the OpenOpt framework [29]. To solve this problem, the parameter *C* (>0) is first arbitrarily chosen. A solver then searches for values of $\beta ={\left(\beta_{1},\ldots ,\beta_{p}\right)}^{T}$, *b* and $\xi ={\left(\xi_{1},\ldots ,\xi_{n}\right)}^{T}$ such that the objective function in (7) is minimized while the constraints in (8), (9) and (10) are satisfied simultaneously. It can be seen that an optimal solution to (7) always exists since the search space of {***β****b****ξ***} is never empty. To find a feasible solution, one can arbitrarily pick $\beta \neq 0\in R^{p}$ and *b* ∈*R* . For *s*_(*i*)_∈*P* , one can pick *ξ*_*i*_∈*R* such that the constraint in (8) is satisfied. Similarly, for *s*_(*i*)_∈*N* , one can pick *ξ*_*i*_∈*R* such that the constraint in (9) is met. We can then compute the value of the objective function as the values of all the variables are known. One way to choose the parameter *C* in (7) is to search for *C* in a range by cross-validation. The parameter is TF-dependent in general, but experiments showed that a small *C* =2−^6^ will usually suffice and hence we set *C* =2−^6^for all the ODV experiments in this study.

### The PSSM and ULPB methods

We briefly describe the ungapped likelihood under positional background (ULPB) method proposed in [23] and the position-specific scoring matrix (PSSM) method compared therein. We refer readers to section Notation for functions and variables used here. Consider a specific TF with binding sites of length *l* . The PSSM method scores an *l* -mer *s* by

(11) $$\overset{l}{\underset{i=1}{\sum }}\text{log}\left[f_{i}(s_{i})\right],$$

where *s*_*i*_ denotes the *i*^th^ letter in *s* . We note that usually the ratio *f*_*i*_(*s*_*i*_)/*f* (*s*_*i*_) is used in place of *f*_*i*_(*s*_*i*_), where *f* (*s*_*i*_) is the background probability of *s*_*i*_. The simpler form in (11) was compared in [23] and hence it serves as a baseline method in this study.

The ULPB models a TFBS by a first-order Markov chain and models the background by another first-order Markov chain. The background transition probabilities are estimated using the entire genome of a species and hence the ULPB method uses negative examples implicitly. It scores an *l* -mer *s* by

(12) $$\text{log}\;f_{1}(s_{1})+\overset{l-1}{\underset{i=1}{\sum }}\text{log}\;\left(\frac{f_{i}(s_{i+1}|s_{i})}{f(s_{i+1}|s_{i})}\right).$$

Although ULPB does not consider background probability in the first term of (12), the score is approximately the log-likelihood ratio of the two Markov chains.

The main difference between the PSSM method and the NPV, ODV and ULPB methods is that the PSSM method does not score nucleotide pairs nor does it utilize a background distribution. The NPV and ODV methods explicitly take advantage of negative binding sites, while the ULPB method does it implicitly by using a background distribution. The flexibility of the proposed framework allows the NPV and ODV methods to easily search in subspaces, further distinguishing the PSSM and ULPB methods from the proposed ones.

## Results and discussion

### Performance assessment and evaluation metrics

The performance of a TFBS search method is evaluated by *ν* -fold cross-validation (CV). Consider a TF with *n*_+ _TFBSs of length *l* with flanking regions on both sides. A set of negative examples, *N*_test_, called the *test negatives* is constructed from the TFBSs of the other TFs with filtering as in [22]. Another set of negative examples, *N*_train_, called the *training negatives* is collected from sequences embedding the *n*_+_binding sites. It is comprised of all the *l* -mers except for the TFBSs and two neighboring *l* -mers of each TFBS.

The *n*_+_ TFBSs are first divided into *ν* sets, each of which contains $\left\lfloor \frac{n_{+}}{\nu }\right\rfloor$ or $\left\lfloor \frac{n_{+}}{\nu }\right\rfloor$ + 1 TFBSs. At each iteration of *ν* -fold CV, one of the *ν* TFBS sets called the *test TFBS setP*_test_is left out. The rest of the TFBSs are therefore called the *training TFBSs* . A scoring function is obtained using the training TFBSs and non-TFBSs randomly sampled from the training negatives, where the ratio of numbers of non-TFBSs to TFBSs is set to 10. The test TFBSs in *P*_test_ along with the non-TFBSs in *N*_test_are then scored by the scoring function. To score a test sequence, both the forward and reverse strands are scored and, in case the test sequence is longer or shorter than *l* , the *l* -mer producing the highest score is used. For each test TFBS *t* ∈*P*_test_, we find its rank relative to all the non-TFBSs in *N*_test_. Formally, the rank of *t* equals 1 + |{*s* ∈* N*_test_|Score(*s* ) ≥ Score(*t* )}|.

After the *ν* -fold CV, we end up with *n*_+_ ranks, each of which corresponds to a TFBS. To allow comparison of methods, we use the area under the ROC curve (AUC) to gauge the performance of a method on the TF. The ROC curve is a plot of true positive rate (TPR) against false positive rate (FPR), displaying the trade-off between TPR and FPR. We refer readers to [30] for an introduction to this metric. In this study, *ν* =10 for all the CV experiments. For the NPV and ODV methods, the best weight and subspace combination is obtained at each iteration of the *ν* -fold CV. Specifically, another (*ν* −1)-fold CV is performed on the *ν* −1 sets of TFBSs to search for the best combination.

### Prokaryotic transcription factor binding sites

To understand the behavior of search methods on prokaryotic TF binding sites, we conducted 10-fold cross-validation experiments on the 26-TF RegulonDB data set. The proposed NPV and ODV methods were compared to the ULPB method [23]. The PSSM method, considered in [23], was also included for comparison since it served as a simple baseline method.

**Figure 4:**
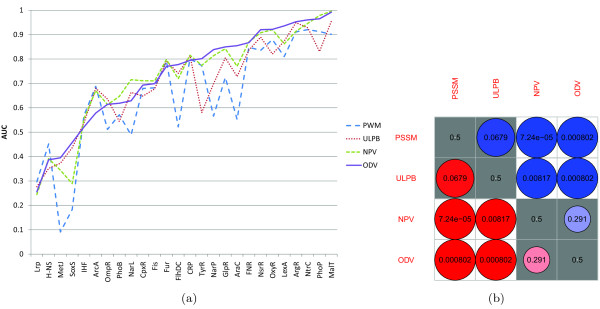
**Comparison of the PSSM, ULPB, NPV and ODV methods on the RegulonDB data set. (a)** Plot of AUC values across the 26 prokaryotic TFs for each method. **(b)** Matrix of *p* -values from pair-wise comparisons. A red solid circle in row *i* and column *j* indicates that method *i* outperformed method *j* , while a blue one in row *i* and column *j* indicates that method *i* is inferior to method *j* . The size and darkness of a circle imply the significance of the relationship between two methods. The larger and darker a circle, the more significant the relationship. White background indicates exceeding the usual 0.05 significance cut-off, while gray background indicates the opposite.

Figure 4a shows the plot of area under the ROC curve (AUC) across the 26 TFs for each method. We can see that the ODV method has the best AUC on 12 out of 26 TFs and the NPV method has the best AUC on 9 out of 26 TFs whereas the ULPB and PSSM methods have the best AUC on 1 and 4 TFs, respectively. To gauge the relative performance between two methods, statistical tests [31] were performed on all the 6 pairs of methods. Figure 4b shows the *p* -values of the pair-wise comparisons. We first notice that, consistent with the results in [23], ULPB outperformed PSSM with a slightly larger *p* -value of 0.0679 than the usual 0.05 significance cut-off. As seen in Figure 4b, the NPV and ODV methods are significantly better than the PSSM and ULPB methods. We can see that the ODV method benefited from optimization albeit minimizing the objective function in (7) does not guarantee maximization of the AUC.

### Eukaryotic transcription factor binding sites

**Figure 5:**
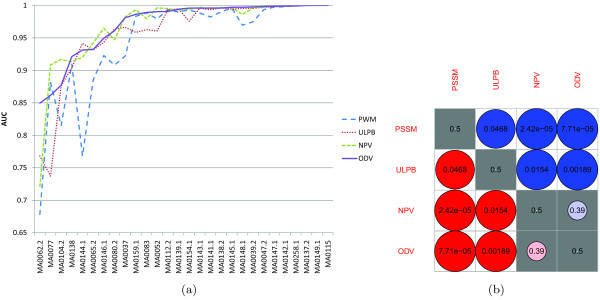
**Comparison of the PSSM, ULPB, NPV and ODV methods on the JASPAR data set. (a)** Plot of AUC values across the 28 eukaryotic TFs for each method. **(b)** Matrix of *p* -values from pair-wise comparisons. A red solid circle in row *i* and column *j* indicates that method *i* outperformed method *j* , while a blue one in row *i* and column *j* indicates that method *i* is inferior to method *j*. The size and darkness of a circle imply the significance of the relationship between two methods. The larger and darker a circle, the more significant the relationship. White background indicates exceeding the usual 0.05 significance cut-off, while gray background indicates the opposite.

Here we compare the proposed NPV and ODV methods to the ULPB and PSSM methods on eukaryotic TF binding sites. As in the previous section, we conducted 10-fold cross-validation experiments on the 28-TF JASPAR data set. Figure 5a shows the plot of AUC across the 28 TFs for each method. We can see that both the ODV and NPV methods have the best AUC on 13 out of 28 TFs while the ULPB and PSSM methods have the best AUC on 6 and 4 TFs, respectively. All the methods have the best AUC of 1 on MA0149.1 and MA0115, while the ODV, NPV and PSSM methods have the best AUC of 0.999 on MA0137.2.

Similarly, statistical tests [31] were performed on all the 6 pairs of methods. Figure 5b shows that the NPV and ODV methods are significantly better than the PSSM and ULPB methods. ULPB is significantly better than PSSM, which is again consistent with the results reported in [23]. Overall, performance of the four methods remain unchanged as we shift from prokaryotic transcription factors to eukaryotic ones. This implies that a TFBS search method effective on prokaryotic transcription factors will perform equally well on eukaryotic transcription factors and vice versa.

### Motif subtype identification in vector spaces

It has been shown that the binding sites of a TF can be better represented by 2 motif subtypes than by a single motif [32,33]. In search for new binding sites, two position-specific scoring matrices are used to score an *l* -mer and the higher score of the two is assigned to this *l* -mer. Searching with two PSSMs was shown to be superior to searching with a single PSSM by cross-species conservation statistics in these studies.

**Figure 6:**
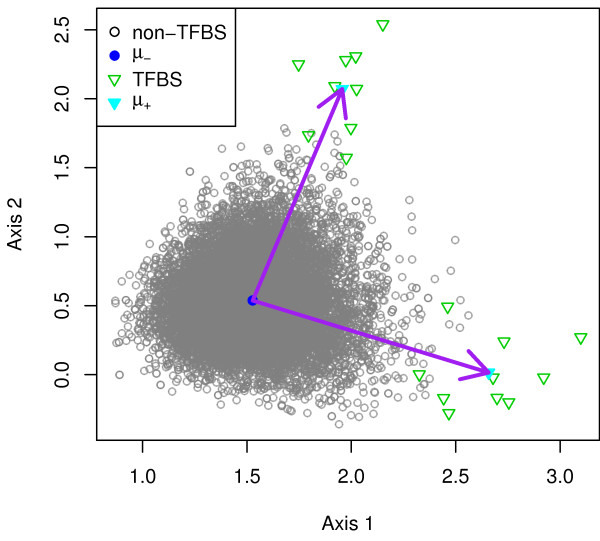
**Illustration of the *k*NPV method.** The solid arrows represent the negative-to-positive vectors ***μ***_+ 1_−***μ***_−_and ***μ***_+ 2_−***μ***_−_, pointing from ***μ***_−_to ***μ***_+ 1_and ***μ***_+ 2_, respectively. The hallow triangles denote the known binding sites, whereas the circles represent the known non-binding sites. The centers of the binding site vectors are marked by the solid triangles, while the center of the non-binding site vectors is marked by the solid circle.

We demonstrate that motif subtypes can be readily identified once we embed *l* -mers in a vector space. The purpose here, however, is not to compare motif subtype identification algorithms. We adopted a slightly different approach to motif subtype identification from those in previous work [32,33], while the idea is similar. As usual, all the *l* -mers were first embedded in a vector space. The known binding sites of a TF were clustered into two subtypes by the *k* -means algorithm [34]. Immediately, we have a variant of the NPV method called the *k* NPV method, where *k* =2 denotes the number of motif subtypes. The *k* NPV method first computes the mean vectors of these two subtypes, ***μ***_+ 1_ and ***μ***_+ 2_, and scores an *l* -mer *s* by

(13) $$\text{Score}(s)=\text{max}\;\left\{s^{T}\left(\mu_{+1}-\mu_{-}\right),s^{T}\left(\mu_{+2}-\mu_{-}\right)\right\},$$

where ***μ***_−_ is the mean vector of the non-binding sites. Figure 6 illustrates the *k* NPV method.

Similarly, the *k* ODV method scores an *l* -mer *s* by

(14) $$\text{Score}(s)=\text{max}\;\left\{s^{T}\beta_{+1}/||\beta_{+1}||,s^{T}\beta_{+2}/||\beta_{+2}||\right\},$$

where ***β***_+ *i*_ is obtained using TFBSs in cluster *i* , *i* =1,2. Unlike the *k* NPV method, the lengths of ***β***_+ *i*_’s may be very different and hence ***β***_+ *i*_’s are scaled to unit vectors so as not to bias the scoring function. We note that the choice of *k* =2 came from previous studies [32,33]. Generally, *k* can be greater than 2 or even automatically selected [35]. This however is beyond the scope of this study and may be investigated in the future.

**Figure 7:**
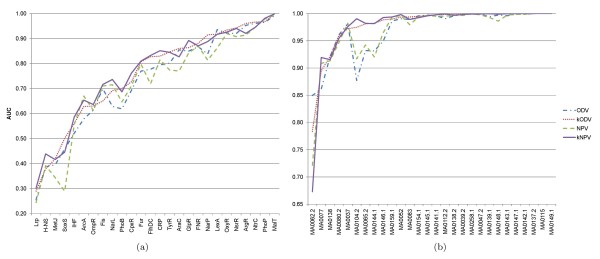
**The *k *NPV (*k* ODV) method versus the NPV (ODV) method.** The number of motif subtypes *k* is set to 2. **(a)** Plot of AUC values across the 26 prokaryotic TFs for each method. **(b)** Plot of AUC values across the 28 eukaryotic TFs for each method.

We assessed the *k* NPV and *k* ODV methods by 10-fold cross-validation on both the RegulonDB and JASPAR data sets. Figure 7 shows the results in terms of AUC. We observe in Figure 7a that overall introducing motif subtypes into the NPV and ODV methods improves the search performance (*p* -values: 6.41 × 10−^7^ and 8.31 × 10−^5^, respectively). Results in Figure 7b also support this observation (*p* -values: 1.61 × 10−^3^ and 3.04 × 10−^3^, respectively). The *k* NPV and *k* ODV are comparable on both the RegulonDB and JASPAR data sets (*p* -values: 0.197 and 0.47, respectively). These results are consistent with those reported in [32,33].

### Independent validation on ChIP-seq data

To evaluate the proposed NPV and ODV methods on the whole genome scale, we built TF models using TFBSs in the JASPAR database to scan all the human (build hg19) 1000-base promoter sequences obtained from the UCSC Genome Browser database [36]. ChIP-seq peaks from the ENCODE project were also retrieved [37]. Specifically, the wgEncodeRegTfbsClusteredV2 table of build hg19 was obtained. We checked TFs in Table 2 against the annotations and found 14 JASPAR TFs, recognized by 17 antibodies present in the ENCODE annotations. The mapping is listed in the first 3 columns of Table 3.

**Table 3:** Results of independent validation on ChIP-seq data

**ENCODE**	**JASPAR**	**Name**	**PSSM**	**ULPB**	**NPV**	**S**	**IC**	**ODV**	**S**	**IC**
GATA3_(SC-268)	MA0037	GATA3	0.48922	0.46841	0.50963	1	Y	**0.51441**	1	Y
MEF2A	MA0052	MEF2A	0.42566	**0.45955**	0.35283	3	Y	0.34807	3	N
PU.1	MA0080.2	SPI1	0.50631	0.49267	0.57575	3	Y	**0.58014**	3	N
SRF	MA0083	SRF	0.34299	0.38457	**0.43920**	2	N	0.43183	3	N
NRSF	MA0138	REST	**0.50615**	0.46371	0.46603	1	N	0.47956	2	N
	MA0138.2	REST	0.48031	0.48299	0.49070	3	Y	**0.49522**	3	N
ERalpha_a	MA0112.2	ESR1	**0.53980**	0.49058	0.52414	3	N	0.52146	1	N
STAT1	MA0137.2	STAT1	0.55348	0.58555	0.61733	1	N	**0.62338**	1	Y
CTCF	MA0139.1	CTCF	0.60370	0.60377	0.63785	2	Y	**0.64769**	2	Y
CTCF_(C-20)			0.44108	0.44696	0.53181			**0.54306**		
CTCF_(SC-5916)			0.46729	0.47047	0.54097			**0.55028**		
FOXA1_(C-20)	MA0148.1	FOXA1	0.48083	0.48698	0.48994	3	Y	**0.49853**	3	N
FOXA1_(SC-101058)			0.48897	0.48326	0.49945			**0.50986**		
EBF	MA0154.1	EBF1	0.50011	0.51202	0.56084	3	Y	**0.59172**	3	N
EBF1_(C-8)			0.42214	0.43705	0.52067			**0.53207**		
FOXA2_(SC-6554)	MA0047.2	Foxa2	**0.48328**	0.39496	0.45500	3	Y	0.47906	3	N
STAT3	MA0144.1	Stat3	0.39145	0.33052	0.38094	3	Y	**0.43807**	3	Y
POU5F1_(SC-9081)	MA0142.1	Pou5f1	0.42151	0.42793	0.40855	3	N	**0.45449**	3	N

Subspaces (S) $R^{4l}$, $R^{\left(16l-12\right)}$ and $R^{\left(36l-48\right)}$ are denoted by 1, 2 and 3, respectively.

For the NPV and ODV methods, the best weight and subspace combination was found by 5-fold cross-validation on the JASPAR TFBSs, while flanking genomic sequences of the TFBSs were the sources of negative binding sites. To assess the 4 compared methods, we considered the part of a ROC curve where FPR is at most 0.01 and calculated the AUC scaled to between 0 and 1. This is nearly equivalent to allowing at most 10 false positive hits per promoter on average. As a peak spans about 200 bases, it is considered recalled when it fully contains a predicted binding site. Similarly, a predicted binding site must be fully covered by a peak to be a true positive hit.

In Table 3, we observe that ODV, NPV, ULPB and PSSM produced the best AUC on 13, 1, 1 and 3 out of 18 tests, respectively. Statistical tests showed that ODV significantly outperformed the other 3 methods (*p* -values ≤ 0.0028), NPV significantly outperformed ULPB and PSSM (*p* -values ≤ 0.0449), and ULPB and PSSM are comparable (*p* -value: 0.433). We notice that both NPV and ODV performed worse than the other two methods on MEF2A. As NPV and ODV both sample negative examples from flanking sequences of TFBSs, we suspect that this is one example where the flanking sequences do not represent well the entire promoters. ODV performed consistently across tests corresponding to the same JASPAR ID such as the three for CTCF. Examining the best weight and subspace, we can see that the subspace agrees on 11 out of 14 TF models, while the weight agrees on only 7 of them. The latter may be because ODV optimizes the ***β*** vector and hence is less sensitive to the weight used to embed an *l* -mer.

## Conclusions

In this work, we proposed to search for transcription factor binding sites in vector spaces. The novel NPV and ODV methods were introduced to construct a query vector to search for binding sites of a TF. We compared our methods to a state-of-the-art method, the ULPB method, and the widely-used PSSM method. Cross-validation experiments revealed that the NPV and ODV methods significantly outperformed the ULPB and PSSM methods on prokaryotic as well as eukaryotic TF binding sties. Independent validation on human ChIP-seq data further verified that the NPV and ODV methods are significantly better than the other compared methods.

One of the advantages of our framework is that it allows one to easily search for binding sites in various subspaces. Hence, one can search in the best subspace for each individual TF since one can hardly find an optimal subspace for all the TFs. Another advantage is that under the proposed framework one can readily identify motif subtypes for a TF. Hence, to exploit this advantage, we introduced the *k* NPV and *k* ODV methods, immediate variants of the NPV and ODV methods. We demonstrated that, consistent with results in previous studies, *k* NPV (*k* ODV) significantly improved NPV (ODV) on the two data sets.

Our future work aims for extending our proposed methods to handling known binding sites of variable lengths. We will seek to approach this problem without resorting to multiple sequence alignment, which is notoriously time-consuming. In the meantime, we will also seek to identify additional promising subspaces to search for TF binding sites in.

## Competing interests

Both authors declared that they have no competing interests.

## Author’s contributions

CL and CH conceived the study. CL collected the data, carried out the experiments and drafted the manuscript. CH guided the study and revised the manuscript. Both authors read and approved the final manuscript.

## Supplementary Material

Additional file 1 Detailed notation. Click here for file
