# Supplementary material for: Searching for transcription factor binding sites in vector spaces
Source: BMC Bioinformatics. 2012 Aug 27;13:215. doi: 10.1186/1471-2105-13-215 (PMC3543194; doi:10.1186/1471-2105-13-215)
Supplement: Additional file 1 — Detailed notation. [file 1471-2105-13-215-S1.pdf]

Detailed notation

- l denotes the binding site length of a transcription factor.
- $f(u)$ denotes the background probability of observing letter u, where $u \in \{\text{A, C, G, T}\}$.
- $f_i(u)$ denotes the probability of observing letter u at position i of a TFBS, where $u \in \{\text{A, C, G, T}\}$ and $i \in \{1, 2, \ldots, l\}$.
- $f(u, v)$ denotes the background probability of observing adjacent letters u and v, where $u, v \in \{\text{A, C, G, T}\}$.
- $f_{i,j}(u, v)$ denotes the probability of observing letters u and v at positions i and j, respectively, where $1 \leq i < j \leq l$ and $u, v \in \{\text{A, C, G, T}\}$. E.g., $f_{2,4}(\text{A, C})$ gives the probability of observing binding sites `*A*C****`, where `*` is the wild card letter representing any DNA letter and the length of TFBSs $l = 8$.
- $f_i(v|u)$ denotes the position-specific conditional probability of observing v at position $i + 1$ given u has been seen at position i, where $u, v \in \{\text{A, C, G, T}\}$ and $i \in \{1, 2, \ldots, l - 1\}$. By definition, $f_i(v|u) = f_{i,i+1}(u, v)/f_i(u)$.
- $f(v|u)$ denotes the background conditional probability of observing v given u has been observed at the previous position, where $u, v \in \{\text{A, C, G, T}\}$. By definition, $f(v|u) = f(u, v)/f(u)$.
- $\mathcal{I}_u(\cdot)$ is the indicator function given by

$$\mathcal{I}_u(v) = \begin{cases} 1 & \text{if } v = u, \\ 0 & \text{otherwise,} \end{cases}$$

  where $u, v \in \{\text{A, C, G, T}\}$. $\mathcal{I}_u(\cdot)$ is used to convert a DNA letter into 4 binary variables. Let $s =$ `CGTAAAACG`. $\mathcal{I}_{\text{A}}(s_1) = 0$ because the first letter of s is not A.
- $\mathcal{I}_{u_1u_2}(\cdot)$ is similarly defined as follows:

$$\mathcal{I}_{u_1u_2}(v_1v_2) = \begin{cases} 1 & \text{if } v_1 = u_1 \text{ and } v_2 = u_2, \\ 0 & \text{otherwise,} \end{cases}$$

  where $u_1, u_2, v_1, v_2 \in \{\text{A, C, G, T}\}$. $\mathcal{I}_{u_1u_2}(\cdot)$ is used to convert a pair of DNA letters into 16 binary variables. Let $s =$ `CGTAAAACG`. $\mathcal{I}_{\text{CT}}(s_1s_3) = 1$ because the first and third letters of s are C and T, respectively.
- IC_i denotes the information content at position i of a binding site. Information content is closely related to entropy, a measure of uncertainty in information theory. The entropy at position i is given by $E_i = -\sum_{u\in\{\text{A, C, G, T}\}} f_i(u) \log_2 [f_i(u)]$. When $f_i(u) = \frac{1}{4}$ for all $u \in \{\text{A, C, G, T}\}$, E_i attains the maximal entropy of 2 and we are most uncertain about the letter at position i. IC_i is simply defined as

$$IC_i = 2 - E_i = 2 + \sum_{u\in\{\text{A, C, G, T}\}} f_i(u) \log_2 [f_i(u)],$$

  where $i \in \{1, 2, \ldots, l\}$.
- $IC_{i,j}$ denotes the information content of the position pair (i, j) of a binding site. Similarly,

$$IC_{i,j} = 4 + \sum_{u,v\in\{\text{A, C, G, T}\}} f_{i,j}(u, v) \log_2 [f_{i,j}(u, v)],$$

  where $1 \leq i < j \leq l$ and the maximal entropy of 4 is attained when $f_{i,j}(u, v) = \frac{1}{16}$ for all $u, v \in \{\text{A, C, G, T}\}$.

- w_i denotes the weight on position i. Two weighting schemes are considered in this study. One sets $w_i = 1$ while the other one sets $w_i = IC_i$, $i = 1, 2, \ldots, l$.
- $w_{i,j}$ denotes the weight on position pair (i, j). Two weighting schemes are considered in this study. One sets $w_{i,j} = 1$ while the other one sets $w_{i,j} = IC_{i,j}$, $1 \leq i < j \leq l$.
- $C > 0$ is a parameter in the optimal discriminating vector (ODV) method. It is fixed to a small constant of 2^{-6} in this study.
